# Herpetic Whitlow Associated With an Eating Disorder: A Case Report

**DOI:** 10.7759/cureus.48654

**Published:** 2023-11-11

**Authors:** Fawaz H Aljehani, Abdullah M Alharthi, Amal W AlDoboke, Ammar A Bakhsh, Amjad Z Alharbi

**Affiliations:** 1 Dermatology, King Abdulaziz Hospital, Makkah, SAU; 2 Medicine and Surgery, Umm Al-Qura University, Makkah, SAU; 3 Medicine and Surgery, Ibn Sina National College, Jeddah, SAU

**Keywords:** fingertip infection, bulimia nervosa, herpes simplex virus, eating disorders, herpetic whitlow

## Abstract

Herpetic whitlow is a localized cutaneous viral infection primarily affecting the fingers, caused by herpes simplex virus types 1 (HSV-1) or 2 (HSV-2). It can be recurrent due to behavioral factors associated with eating disorders, such as bulimia nervosa. We would like to introduce “Jehany Sign,” suggesting the term for the association of bulimia nervosa with HSV infections. Here, we present the case of a 50-year-old pre-diabetic female with recurrent herpetic whitlow on her left index finger associated with self-induced vomiting and extreme concern about her weight and body shape. Skin examination showed an eroded group of vesicles on an erythematous base on her left finger. The diagnosis was established through history and clinical examination. Upon follow-up, the patient showed complete resolution in one week after receiving topical and systemic acyclovir, which led to a subsequent referral to a psychiatrist for further management regarding bulimia nervosa. This case highlights the importance of a multidisciplinary approach and the complicated connections between eating disorders and dermatological diseases. Recognizing these allows healthcare providers to deliver more comprehensive care, improve patient outcomes, and further study in this area.

## Introduction

Stern et al. initially described herpetic whitlow in 1959 [1]. Herpetic whitlow is an infection frequently caused by type 1 or 2 herpes simplex virus (HSV) [1]. It is recognized by a prodrome consisting of a burning feeling in the fingers, which is then followed by erythema, pain, and the development of non-purulent vesicles [2]. Herpetic whitlow is expected to impact 2.5 out of 100,000 people annually [3]. This is one of the numerous reasons why the disease can sometimes be misdiagnosed or underdiagnosed [3]. Herpetic whitlow is more likely to occur once an individual has been in contact with oral secretions [4]. Bulimia nervosa is a disorder manifest by compensatory behaviors, including self-induced vomiting and the misuse of laxatives and diuretics, which pose a serious risk to a person’s life and can be challenging to diagnose due to their relationship with a wide variety of other mental health conditions [5,6]. Thus, continuous exposure to oral secretion increases the risk of herpetic whitlow. Therefore, acquiring a comprehensive history is a crucial part of the diagnostic process. This case demonstrated that even in immunocompetent patients, herpes whitlow could recur for various reasons; in this case, the patient’s bulimia nervosa led her to put her finger in her mouth to induce vomiting repeatedly. Understanding the clinical signs and carefully recording past medical history will help prevent the use of unnecessary pharmaceuticals, especially in cases with recurring lesions. Until lesions have healed, dermatologists may be advised to avoid direct patient contact.

## Case presentation

A 50-year-old pre-diabetic female patient on metformin 500 mg presented to the dermatology clinic with symptomatic, recurrent painful vesicles on her left index finger for a long period. These lesions were typically associated with repeated episodes of excessive food consumption followed by self-induced vomiting. She had an extreme concern about her weight and body shape. Her past medical history was remarkable for recurrent on and off lesions on her left index finger, though she was never diagnosed, and no medication was given to her before. She had no history of oral or genital herpetic infection. There was no significant weight loss and no past surgical history. She was not using any medication for weight loss.

**Figure 1:**
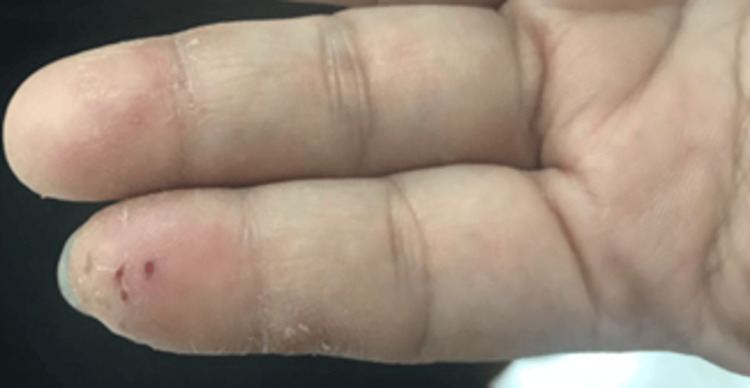
Clinical manifestation before treatment. Eroded group of vesicles on an erythematous base over the left index finger.

Her general examination upon presentation showed stable vital signs, and the remainder of her examination was unremarkable. Skin examination revealed the characteristic grouped vesicles, some of which were eroded on a background of erythema on her left index finger (Figure 1).

Polymerase chain reaction and biopsy were not performed. Based on the previous clinical findings and past medical history of continuously putting her finger in her mouth to vomit, she was diagnosed with recurrent herpetic whitlow.

**Figure 2:**
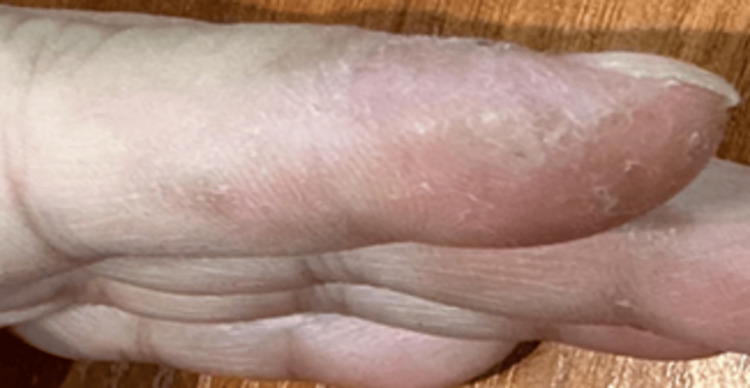
Clinical manifestation after treatment. The lesion significantly showed complete resolution.

As in our case, many healthcare professionals are unsure of the connection between eating disorders and HSV infections, leading to incorrect diagnoses. We believe that “Jehany Sign” is an appropriate term to refer to any psychiatric condition associated with an HSV infection. The patient was concerned about the diagnosis but was reassured and started on topical and systemic acyclovir and was referred to psychiatry to treat her primary eating disorder. Upon follow-up, with compliance with the treatment, complete resolution was observed after one week (Figure 2).

## Discussion

Herpetic whitlow is a painful cutaneous viral infection that most commonly affects the distal phalanx of the fingers and occasionally the toes. It is caused by HSV types 1 or 2. It occurs mainly in adults aged 20 to 30 years and children. In children, most cases can be attributed to autoinoculation of HSV-1, while in adolescents and adults, herpetic whitlow tends to be caused by autoinoculation of HSV-2. Herpetic whitlow may have a prodrome of burning, pruritus, and/or tingling of the affected finger or the entire limb, followed by erythema, pain, and vesicle formation [2]. HSV spreads by direct digital contact with secretions from lesions of infected patients or asymptomatic carriers. About 20% to 50% of affected patients experience recurrence that may be triggered by illness, fever, sun exposure, menstruation, or other physiological or psychological stressors [1].

Bulimia nervosa has a considerable negative impact on each of the body’s major organs. One of the affected organs is the skin. Women are increasingly more likely than men to have eating disorders. One study comparing the restrictive and bulimic forms of anorexia nervosa found that patients with the bulimic form had more specific skin abnormalities, such as seborrheic dermatitis, Russell’s sign, acne, nail changes, generalized pruritus, and hair effluvium [7].

There is a long-standing recognition that eating disorders have a significant impact on various aspects of health, including dermatologic conditions. Comprehensive research in the field has shed light on the multiple disorders that can be linked to these conditions. However, it is important to note that HSV viral infection recently emerged as having a potential association with eating disorders. Conditions such as dry skin, brittle nails, acne, generalized pruritus, and hair loss have been commonly observed in individuals with eating disorders. Bulimic anorexia nervosa is characterized by Russell’s sign, which describes knuckle calluses on the dorsal side of the dominant hand from teeth contact of the patient’s hand with the mouth during self-induced vomiting. These physical manifestations often reflect the underlying nutritional deficiencies and imbalances that accompany these disorders. Researchers have hypothesized that the weakened immune system and compromised nutritional status commonly seen in individuals with eating disorders may contribute to an increased susceptibility to HSV infections [8-10].

The strength of our case is the finding that, even when the diagnosis is clear, physicians should always consider alternative reasons and take a detailed medical history to optimize the patient’s medical outcome because treating the underlying cause will prevent disease recurrence. Our patient was getting better on acyclovir. It is possible to diagnose an eating disorder simply by conducting a thorough examination of the skin. Dermatologists, the majority of whose patients are female, have the distinct advantage of being in a position to identify these signs. This is highly essential because the prognosis will be affected by how early a diagnosis is made. After discussing her symptoms with a psychiatrist, it was concluded that the patient had bulimia nervosa. A limitation of our case is the lack of reported cases of herpetic whitlow due to psychiatric illnesses in the literature.

To raise awareness that HSV can manifest as a primary eating disorder, we would like to suggest the term “Jehany Sign,” which is defined as recurrent, painful deep pustules that get eroded and dried on the plantar aspect of the finger on the dominant hand due to the association with self-induced vomiting over long periods of time. The condition develops when the patient repeatedly inserts their finger into the oral cavity and is exposed to vomits and oral secretions while triggering the gag reflex at the back of their throat, regardless of any prior exposure to the herpes virus.

This case demonstrates how challenging it is to diagnose recurrent herpetic whitlow when it is related to psychological problems.

## Conclusions

This case demonstrates the challenge of diagnosing recurrent herpetic whitlow induced by self-induced vomiting in a patient with bulimia nervosa. Our findings highlight the crucial role of a thorough medical history and consideration of underlying causes in dermatological diagnoses. Successful treatment with acyclovir, combined with psychiatric assessment, highlights the importance of addressing the cause for preventing disease recurrence. Furthermore, our case suggests a potential link between HSV infections and bulimia nervosa, leading us to introduce the term “Jehany Sign.” This concise term summarizes the manifestation of herpetic whitlow in the context of bulimia nervosa. This particular case emphasizes the importance of a multidisciplinary approach and the complicated connections between mental health and dermatological disorders. Recognizing these allows healthcare providers to deliver more comprehensive care, improve patient outcomes, and further investigation in this area.
